# [Corrigendum] Anti‑osteoporotic effects of tetramethylpyrazine via promoting osteogenic differentiation and inhibiting osteoclast formation

**DOI:** 10.3892/mmr.2023.13061

**Published:** 2023-07-31

**Authors:** Long Wang, Wei-Guang Lu, Jun Shi, Hong-Yang Zhang, Xiao-Long Xu, Bo Gao, Qiang Huang, Xiao-Jie Li, Ya-Qian Hu, Qiang Jie, Zhuo-Jing Luo, Liu Yang

Mol Med Rep 16: 8307–8314, 2017; DOI: 10.3892/mmr.2017.7610

Subsequently to the publication of the above paper, an interested reader drew to the authors’ attention that, in Fig. 2A on p. 8311, portraying the results of immunostaining experiments for osterix, the ‘GIOP’ and ‘GIOP+TMP (20)’ data panels contained overlapping data, such that these images were derived from apparently the same original source, where they were intended to show the results from differently performed experiments. Moreover, in Fig. 3A on p. 8312 showing the results from ALP staining and Alizarin Red S staining experiments, two pairs of apparently overlapping data panels were identified in the Dex 10^6^ M / TMP 50 μM, 100 μM and 200 μM data panels.

After having re-examined their original data, the authors have realized that the data featured in Figs. 2A and 3A were assembled incorrectly in these figures. Revised versions of Fig. 2 and 3, now containing replacement data for the experiments shown in Figs. 2A and 3A, are shown on the next page. Note that these errors did not adversely affect either the results or the overall conclusions reported in this study. All the authors agree with the publication of this corrigendum, and are grateful to the Editor of *Molecular Medicine Reports* for allowing them the opportunity to publish this. They also wish to apologize to the readership of the Journal for any inconvenience caused.

## Figures and Tables

**Figure 2.**
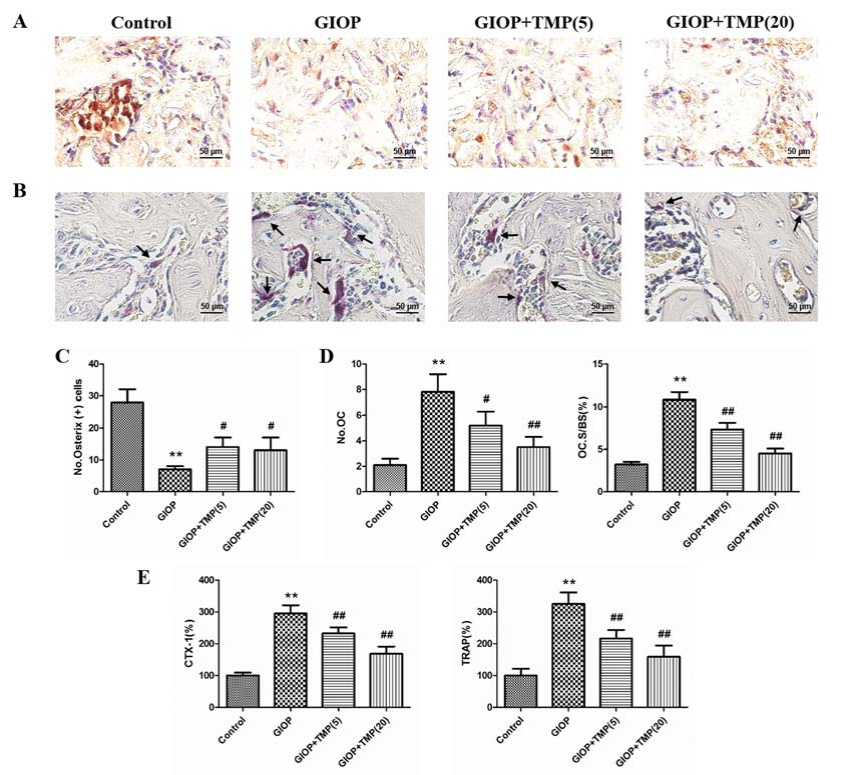
TMP promotes osteogenesis and inhibits osteoclastogenesis *in vivo*. (A) Representative staining (brown) of osterix. (B) TRAP staining of distal femurs. Arrows indicate areas of TRAP-positive multinuclear cells. (C) Quantification of osterix-positive cells. (D) TRAP-positive multinuclear (≥3) cells of each group were counted. (E) Serum levels of CTX-1 and TRAP in rats. The control value of CTX-1 was 25.71±2.37 ng/ml and the control value of TRAP was 19.36±2.55 U/ml. **P<0.01, vs. control group; ^#^P<0.05 and ^##^P<0.01, vs. GIOP group (n=5). (5) and (20) represent 5 and 20 mg/kg body weight of TMP, respectively. GIOP, glucocorticoid-induced osteoporosis; TMP, tetramethylpyrazine; OC, osteoclasts; OC.S/BS, osteoclast surface/bone surface; TRAP, tartrate-resistant acid phosphatase.

**Figure 3.**
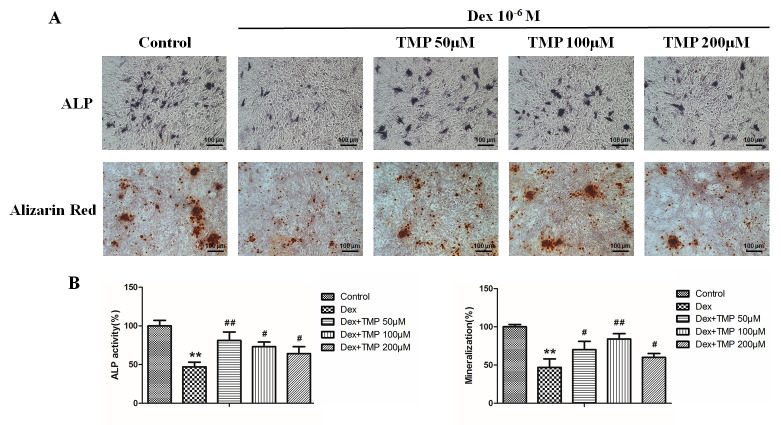
TMP promotes osteogenic differentiation of BMSCs in the presence of excess glucocorticoids *in vitro.* (A) Images of ALP staining and Alizarin Red S staining. (B) Activity of ALP and mineralization of BMSCs were calculated. The control value for the activity of ALP in BMSCs was 0.49±0.06 U/mg protein. **P<0.01 vs. the control group; ^#^P<0.05 and ^##^P<0.01 vs. the Dex group (n=5). ALP, alkaline phosphatase; Dex, dexamethasone; TMP, tetramethylpyrazine.

